# Supplementary material for: Impact of vitamin C on the reduction of opioid consumption for acute musculoskeletal pain: A double-blind randomized control pilot study
Source: PLoS One. 2024 Dec 31;19(12):e0316450. doi: 10.1371/journal.pone.0316450 (PMC11687786; doi:10.1371/journal.pone.0316450)
Supplement: S1 Table — (DOCX) [file pone.0316450.s001.docx]

Supplemental Table 1. Comparison of baseline characteristics between participants who were compliant and those who were not.

| Baseline characteristics | Compliant (N=33) | Non-compliant (N=7) |
| --- | --- | --- |
| Mean (±SD) age | 51.5 (14.8) | 58.6 (20.3) |
| Female (%) | 45.5 | 42.9 |
| Mean (±SD) pain intensity (0-10 scale) at triage | 8.1 (1.8) | 8.1 (1.8) |
| Type of pain conditions (%)  -Fracture  -Contusion  -Back pain  -Neck pain  -Other musculoskeletal pain | 42.4  9.1  33.3  6.1  9.1 | 28.6  0  28.6  14.3  28.6 |
| Treated with opioid within the ED stay (%) | 51.5 | 57.1 |
| Median (Q1-Q3) ED stay (hours) | 5.3 (3.8-9.3) | 4.7 (3.1-7.2) |

Q1-Q3: first and third quartile; ED: emergency department; SD: standard deviation
