# Supplementary material for: Impact of vitamin C on the reduction of opioid consumption for acute musculoskeletal pain: A double-blind randomized control pilot study
Source: PLoS One. 2024 Dec 31;19(12):e0316450. doi: 10.1371/journal.pone.0316450 (PMC11687786; doi:10.1371/journal.pone.0316450)
Supplement: Attachment — Submitted filename: Response_to_Reviewers_15_09_2024_RD.docx [file pone.0316450.s002.docx]

September 16, 2024

**Revision for manuscript:** ID: PONE-D-24-19288

Journal:  PLOS ONE

Title: Impact of Vitamin C on the Reduction of Opioid Consumption for Acute Musculoskeletal Pain: A Double-Blind Randomized Control Pilot Study

Axel Benhamed, M.D, MSc
Academic Editor
PLOS ONE

Dear Dr Benhamed,

We would like to thank you for giving us the opportunity to resubmit a revised version of the manuscript. The co-authors and I have reworked the manuscript following the editor and reviewers’ recommendations. All comments and suggestions have been considered in our revisions and changes to the manuscript have been made using track changes. We also want to thank the reviewers for their suggestions, which helped us to significantly improve the paper’s quality.

**Academic Editor**

1) I suggest authors to integrate the study rationale into the introduction section of the manuscript;

**Response: Done**. We removed the study rationale and study objectives headings in the introduction section. The study rational is now integrated into the introduction.

**Reviewer: 1**

1) As the statistical reviewer I will focus on methods and reporting. All looks reasonable but I was confused because the outcome for the future trial was not covered. one of the major aims of feasibility outcomes is to assess the outcome of interest for the future trial, obtain estimates of variability and reasonable effectiveness levels - that does not seem to be the case here or I have missed it. A section is needed on the next steps and what information could be used for the future trial, ideally a power calculation towards that.

**Response: Done**. The outcome of interest for the future trial is the median number of morphine 5mg tablets consumed in the two groups and is reported in Table 4. In that table, we reported the median and the 25-75 quartiles for each group. We now added the effect size related to the treatment effect in the results section even if the study was not designed to conclude on the efficacy of the treatment. “For the intention to treat analysis, the median number of morphine 5mg tablets consumed was higher in the vitamin C group than the placebo group (8.5 vs 6.0 tablets; *effect size: r = 0.27*). and when we examine the per-protocol analysis, the median number of morphine 5mg tablets consumed was lower in the vitamin C group than the placebo group (6.5 vs 9.0 tablets; *effect size: r = -0.11*).”

We also added in the statistical analysis section a sentence to explain how we calculate the effect size from median differences: “We calculated the treatment effect size by using the formula r = z/√N, where z comes from the Mann-Whitney U test.”

**Reviewer: 2**

However, the authors should develop their hypothesis at greater length to explain the difference between per-protocol analysis and intention-to-treat analysis, which is theoretically the most appropriate method for analyzing a clinical trial. However, the intention-to-treat analysis, which shows a higher consumption of morphine in the vitamin C group, "in theory", does not really go in the direction intended by the authors. A lack of power cannot be invoked, since the inclusion of additional patients has methodologically no reason to change the direction of the difference. The authors therefore need to explain why they believe that the intention-to-treat analysis of this small feasibility study (which, it is true, is not designed to conclude on the efficacy of the treatment) is not predictive of a result in a future clinical trial.

**Response:** We did address the difference in intention-to-treat and per-protocol results in the limitations section: *“*This pilot RCT was not powered to detect differences in clinical outcomes, so any between-group comparison should be interpreted with caution. The wide confidence intervals and discordant results between intention-to-treat and per-protocol attest to this. We also added that *“Since non-compliance is associated with less pain, particularly in the second week of treatment, this could partly explain the discordant results observed in our small sample.”*

Specific comments:

The authors' assertion associated with reference 25 that vitamin C is a drug with very low toxicity, even if globally acceptable, could be tempered by a recent article on vitamin C and sepsis in intensive care, which was associated in this clinical trial with excess mortality in the vitamin C group.

**Response:** The LOVIT study was done in a very different population and much higher dosage of vitamin C “proven or suspected infection as the main diagnosis, and who were receiving a vasopressor to receive an infusion of either vitamin C (at a dose of 50 mg per kilogram of body weight) … every 6 hours”. This excess of mortality as not been reported at dosage used in our study.

It seems to me that one piece of information is missing from the study's rationale: what is the percentage of opioid prescriptions in musculoskeletal trauma patients in the authors' department where this clinical trial is taking place, based on retrospective data, and what other therapeutic classes are used? These figures could be compared with, if available, similar data from other publications in Canada, the USA or Europe.

**Response:** We agree that this information could be of interest to the reader. However, the information on physician prescription after ED discharge is not enter on our database system. Therefore, that information is not available.

In the paragraph on vitamin C on page 6, the authors could indicate that the doses delivered are far in excess of physiological vitamin C requirements.

**Response: Done**. We added this in the paragraph on vitamin C: *“However, this dosage exceeds the adult Recommended Dietary Allowances set by the Government of Canada, which is 65 to 90 milligrams per day.”*

The authors indicate a median consent rate of 72% and a median retention rate of 88%. Could they clarify the definition of these two rates, as I don't understand why the retention rate is higher than the consent rate, unless the 88% is itself related to patients who have already consented.

**Response:** The 88% relates only to the patients who consented. To clarify this, we changed the phrase *“To assess feasibility, our results were compared to those reported in a recent review of randomized controlled trials; 72% (IQR: 50%–88%) median consent rate of eligible patients and 88% (IQR: 80%–97%) median retention rate of patients who consented.”*

In Table 2, the authors note several important differences between the vitamin C group and the placebo group (sex ratio, paracetamol, etc.). Would it be possible to have a column with the "p's" (as requested for the other tables)? If I've understood correctly, the authors attribute these differences to chance, due to the small number of patients compared?

**Response:** The information in Table 2 and in the other tables is for descriptive purposes only. They are not statistically tested because they are not powered and they are not subject to hypothesis testing.

The level of pain intensity above 7 confirms that this is severe pain, for which the recommendations are indeed to obtain a morphine prescription. However, pain intensity at admission cannot prejudge pain intensity at discharge from the emergency department, especially in patients with immobilization, which is itself highly analgesic. It would therefore be interesting to have the discharge VAS from Table 1.

**Response:** The information in Table 1 compares patients who refused to participate with those who participated in our study. Pain intensity at discharge for patients who refused to participate is not available.

Concerning pain intensity, as shown in figure 3, it can be seen that on average, both in per-protocol analysis and even more so in intention-to-treat analysis, pain was greater by between half and one VAS point each day in the vitamin C group. What was the statistical test used to compare these two series in figure 3? It seems to me that they can be treated as Kaplan-Meier curves.

**Response:** For our main outcome shown in Table 4, pain intensity over the 14 days was reported for descriptive purposes only. In a pilot study, treatment effects are not statistically tested because of power issues and because it is not intended to test treatment effect. We report only the mean and SEM for each group to aid in the design of a future multicenter randomized trial.

**Reviewer: 3**

Some texts in the introduction seem to repeat themselves with slight nuances. For example, on page 3: "Currently, the main strategy employed to reduce opioid consumption is to limit the rate and quantity of opioids prescribed for acute pain. [12-15] However, most interventions reduce opioid prescription rates, but not the quantity of prescribed opioids. [16]"

**Response:** These sentences do not communicate the same thing. The first describes approaches and the second describes the effects of those approaches.

Patient and Public Involvement: please note the reference of ethic comitee.

**Response:** **Done**. We added the name of the ethic committee and the study reference number: *“*This study was approved by the local Ethics Review Committee *(Comité d'éthique de la recherche du CIUSSS du Nord-de-l'Île-de-Montréal: MP-32-2023-2442).”*

Results should only appear in the results section. For example :
"The main barriers were the high refusal rate (41%) by eligible patients, mainly because they lacked interest for the study, were in too much pain and did not have enough time to participate" OR "Our median (IQR) retention rate of 75% is lower than the 88% (80%-97%) reported in the same review"

**Response: Done.** Those numbers are now removed from the discussion section.

To enhance the comprehensiveness of the article, I recommend incorporating a more detailed discussion of the pathophysiology, either in the introduction or the discussion section. This will provide a better understanding of the mechanisms by which vitamin C exerts its effects in this context.

**Response: Done**. In the introduction section we added “There is no consensus as to its analgesic mechanism. The most widely accepted mechanism involves reducing free radicals’ production, which helps protect tissues (including nerves) from irreversible damage. It may also be a cofactor for the biosynthesis of opioid peptides, and modulate the expression of genes involved in pain perception.”

**Reviewer: 4**

So, the main outcome for feasibility was the recruitment rate. This work is very clearly presented and the article is well constructed. However, I fail to understand its purpose and the importance of the main result. Especially as the expected number of patients was not reached, with a high rate of loss to follow-up (25%), giving the opposite impression to that suggested by the authors. In this context, I find it very difficult to have an opinion on the scientific interest of such a publication.

**Response:** The main purpose of this type of study is to inform the design and risk of failure/success of future larger similar trials. If a researcher reads a feasibility study that shows that a full trial on a particular topic would be unlikely to meet recruitment/retention rates and other feasibility outcomes, then the potential wasted cost of that future trial is avoided, or the planned trial design can be modified to avoid the obstacles identified in the feasibility study. On the other hand, if the feasibility study shows that a full trial is likely to be successfully completed, researchers planning similar studies can be assured that the cost and effort are likely to be worthwhile.

We thank you for the review of this manuscript. We hope that our revised version meets your standards, and that the manuscript is now suitable for publication.

Please do not hesitate to contact the undersigned for further information.

Best regards,

Raoul Daoust, MD, MSc

*Corresponding Author*

*Emergency Department*

*Sacre-Cœur Hospital*

*5400 Gouin blvd. West*

*Montreal (Qc) Canada H4J 1C5*

E-mail: raoul.daoust@videotron.ca
